# Effect of Selenium Fortification on Growth Performance and Nutritional Compounds of Kale (*Brassica oleracea* L. Var. *acephala* DC.)

**DOI:** 10.3390/foods14183283

**Published:** 2025-09-22

**Authors:** Xiu-Ying Zeng, Han Liao, Le-Cheng Shen, Qi Zou, Ting-Ting Lv, Mei Wang, Xiao-Yin Wang

**Affiliations:** 1 Ganzhou General Inspection and Testing Institute, China National Quality and Inspection Center for Se-Rich and Camellia Oleifera Products (Jiangxi), Ganzhou 341000, China; shanshuihuscau4966@163.com (X.-Y.Z.); liaohan871109@163.com (H.L.); shenlechen@163.com (L.-C.S.); 2022010004@ecut.edu.cn (T.-T.L.); wangmei84@163.com (M.W.); 2 School of Public Health and Health Management, Gannan Medical University, Ganzhou 341000, China; 3 Key Laboratory of Development and Utilization of Gannan Characteristic Food Function Component of Ganzhou, Gannan Medical University, Ganzhou 341000, China

**Keywords:** Se-enriched vegetable, selenium speciation, bioactive compounds, quasi-targeted metabolomics, correlation

## Abstract

This study aims to investigate the effects of selenium (Se) fortification on growth performance and the Se content in kale using Se fertilizer, and it determines the influences of Se fortification on the metabolic profile of kale using quasi-targeted metabolomics. The results showed that Se fortification increased the plant height and leaf weight of kale, up-regulated the total Se content and decreased the chlorophyll and total phenolic contents in kale leaf. Se fortification elevated selenate (Se(IV)), selenite (Se(VI)), selenocystine (SeCys_2_), Se-methylselenocysteine (Se-MeSeCys) and selenomethionine (SeMet) contents, as well as total contents of Se in different forms in kale leaf. Se fortification also changed the metabolic profile of kale leaf, via six particular types of compounds (amino acid and its derivatives; organic acid and its derivatives; carbohydrates and its derivatives; lipids; flavonoids; organoheterocyclic compounds) and eight metabolic pathways (alanine, aspartate and glutamate metabolism; amino sugar and nucleotide sugar metabolism; sulfur metabolism; starch and sucrose metabolism; taurine and hypotaurine metabolism; glycolysis/gluconeogenesis; fructose and mannose metabolism; nitrogen metabolism). Moreover, 24 metabolic biomarkers were screened for kale leaf affected by Se fortification. Furthermore, correlations were observed between metabolic biomarkers and Se contents as well as speciation. These results indicate that Se fortification has a significant influence on the growth performance and nutritional compounds of kale, providing references for the future study on the production and bioactivity of Se-enriched kale.

## 1. Introduction

Kale (*Brassica oleracea* L. Var. *acephala* DC.), an important vegetable belonging to the *Brassica* family, is commonly consumed in Europe, Asia and America. In recent years, it has gained great popularity as a “superfood” and is listed in many “lists of the healthiest vegetables” [1]. This vegetable is rich in nutrients and bioactive compounds, such as amino acids, vitamins, minerals, dietary fiber and phytochemicals [2]. Kale extracts have been reported to possess numerous preventive and therapeutic properties, such as antimicrobial, anti-carcinogenic, antioxidant and hypoglycemic activities [3]. In view of this, kale is increasingly considered a source of nutraceuticals and undergoes processing to become value-added products (bread, juice, beverages, etc.) [4].

Kale plant is an annual crop, whose yield, size and nutritional variation greatly depend on its growing conditions. The effects of bio-slurry and inorganic N fertilizer [5], sulfur treatment [2], temperature stress [6,7], nitrogen and magnesium nutrients [8], iodoquinoline biofortification [9] and selenium (Se) biofortification [10,11] on the growth performance, nutrients and/or bioactive compounds of kale are some of the areas that have been investigated to date.

It is well known that Se is an essential trace element that plays a crucial role in maintaining animal and human health. Se deficiency is closely related to several diseases such as Kashan, Kashin–Beck and hypothyroidism [12], while its overdose causes intoxication events [13]. Approximately one billion people suffer from Se deficiency globally, as the soil of many countries lacks this element [14]. In fact, the organic compositions of Se species, especially selenoproteins and selenoamino acids, are responsible for the function of Se in human health. Since plants can generally convert inorganic Se into organic Se, the aforementioned Se biofortification has been widely applied to produce Se-enriched foods [12], thereby increasing Se intake in human diets and satisfying the recommended dietary allowance [14]. Like with other *Brassica* plants (turnip, cabbage, broccoli, nozawana, komatsuna, etc.), kale can also significantly absorb, accumulate and transform inorganic Se into organic Se [12]. Thus, kale has great potential to produce as Se-enriched diet.

Se biofortification has demonstrated the ability to enrich the Se content in kale. In a study by Tavan et al. [14], kale microgreens successfully accumulated up to 893.3 and 24 µg Se/kg of dry matter under a Se-rich soilless medium and with foliar application, at a 20 µM concentration. Leamsamrong et al. [10] found that the total Se contents of the air-dried matter in Se-enriched Chinese kale seedlings (433 ± 22 mg·Se/kg) were significantly higher than those of the regular Chinese kale seedlings. Maneetong et al. [12] discovered that total Se concentrations of kale with all Se-supplemented treatments were higher than those with the control treatment. Furthermore, the investigation conducted by Zagrodzki et al. [11] indicated that Se fortification stimulated the production of phenolic acids (sinapic, chlorogenic, isochlorogenic and caffeic acids) in the kale sprouts. Paśko et al. [15] observed that Se fortification affected the synthesis of sulfur and phenolic compounds in the kale sprouts. However, the influences of Se biofortification on comprehensive nutritional compounds in kale are still necessary to examine. Therefore, a comprehensive profiling method is required to elucidate the underlying metabolic changes.

Quasi-targeted metabolomics, based on LC-MS/MS technology, is a new type of metabolomics detection technique that combines the high-throughput advantages of untargeted metabolomics with the high accuracy and sensitivity advantages of targeted metabolomics. This tool has been widely used to identify chemical constituents and metabolic changes in plants [16,17,18,19]. For instance, Ren et al. [20] examined an integrated metabolome to reveal the differential metabolites in a vegetable cucumber fruit (*Cucumis sativus* L.) after grafting. Moreover, other researchers have used an untargeted metabolomics strategy to study the nutrient profiles of Chinese kale [21]. In this light, quasi-targeted metabolomics is suitable for understanding the effect of Se biofortification on nutritional compounds in kale.

Hence, in this study, the effects of Se fortification on growth performance and the Se content in kale were detected. Moreover, the influences of Se fortification on the metabolic profile of kale were systematically determined and analyzed using quasi-targeted metabolomics. Furthermore, correlations between the metabolic biomarkers, Se contents and speciation in kale were analyzed. This study is beneficial for people to better understand the impact of selenium fortification on the nutritional compounds in kale, and it can guide future research on the potential biological activities of Se-biofortified kale.

## 2. Materials and Methods

### 2.1. Materials and Chemicals

Kale seeds were purchased from Beijing Dongsheng Seed Industry Co., Ltd. (Beijing, China). Amino acid water-soluble fertilizer (containing Se ≥ 1000 mg/L) was bought from Suzhou Setek Co., Ltd. (Suzhou, China). A mixed standard solution of 28 metal elements was obtained from the National Center of Analysis and Testing for Nonferrous Metals and Electronic Materials, and standard solutions of Se(IV), Se(VI), SeCys_2_, Se-MeSeCys and SeMet were gained from the National Institute of Metrology, China. Gallic acid was purchased from LEMEITIAN MEDICINE (Chengdu, China), and Folin–Ciocalteu reagent was bought from Shanghai Macklin Biochemical Co., Ltd. (Shanghai, China). LC-MS-grade water was obtained from Merck KGaA (Darmstadt, Germany), and LC-MS-grade methanol, formic acid and acetonitrile were gained from Thermo Fisher Scientific (Waltham, MA, USA). All other chemicals that were used were of analytical grade and bought from Sinopharm Chemical Reagent Co., Ltd. (Shanghai, China).

### 2.2. Experimental Site, Growing Conditions and Cultivation

The experiment was conducted at the test base of the Ganzhou General Inspection and Testing Institute (25.8351° E, 114.8957° N) in Ganzhou City, Jiangxi province, China. Kale seeds were purchased, soaked, germinated and then sown in a tray using a seedling substrate to grow seedlings. One month later, kale seedlings with thick main stems and well-developed root systems, having 3–4 true leaves and showing consistent growth, were selected and transplanted into 100 L vegetable boxes (3 plants in each box). The Se contents of soil in all vegetable boxes were ≤0.4 mg/kg. Three months later, kale plants were divided into six groups (6 plants per group): the control group (treated with 0 mg/L Se); the 1 mg/L Se group; the 2 mg/L Se group; the 5 mg/L Se group; the 10 mg/L Se group; and the 20 mg/L Se group. The Se solutions with different concentrations that were used in this study were prepared using an amino acid water-soluble fertilizer (containing Se ≥ 1000 mg/L). The leaf surface of each plant and the corresponding soil were sprayed with 500 mL of prepared Se solutions for two months. After the experimental period, biometric measurements of kale were taken, and the 4th to 10th kale leaves were collected from top to bottom for the following determinations.

### 2.3. Determination of Biometric Measurements of Kale

The plant height of kale was determined by measuring the distance from the bottom to the top of the stem using a vernier caliper. In addition, all leaves of each kale plant were picked and then weighed using an electronic scale (ME104E, Mettler Toledo, Shanghai, China), and the weight was recorded as leaf weight.

### 2.4. Determination of Chlorophyll Contents in Kale Leaf

The chlorophyll content in kale leaf was detected according to the method recommended by the Standardization Administration of the People’s Republic of China (NY/T 3082-2017) [22]. To achieve this, 0.5 g of kale leaf was placed in a conical flask, and it was mixed with 100 mL of an absolute ethyl alcohol–acetone solution (*v*/*v* = 1:1). The conical flask was sealed with a sealing film, and then the mixture was kept standing in the dark at room temperature for 5 h. After filtration, the absorbances of the filtrates were measured at 645 nm and 663 nm, respectively. Finally, the chlorophyll content was calculated using the Arnon Equation (1):

*w* = (8.05 × *A*_1_ + 20.29 × *A*_2_) × *v*/(1000 × *m*) (1)

where *w* is the chlorophyll content; *A*_1_ and *A*_2_ are the absorbance of the sample at 645 nm and 663 nm, respectively; *v* is the sample volume; and *m* is the sample weight.

### 2.5. Determination of Total Phenolic Contents in Kale Leaf

The total phenolic content in kale leaf was colorimetrically determined using the Folin–Ciocalteu method, referring to previous study [23] with slight modifications. In the process, 0.2 g of kale leaves was weighed and adequately homogenized with 50% methanol (*v*/*v*). The homogenates were centrifuged to collect the supernatants, and 1 mL of the supernatant was mixed with 1 mL of the Folin–Ciocalteu reagent and 2 mL of a Na_2_CO_3_ solution (20 g/L). Then, the mixture was reacted in the dark at room temperature for 2 h. Finally, the absorbance was measured at 765 nm. In this determination, gallic acid was used as the standard.

### 2.6. Determination of Total Se Contents in Kale Leaf

The total Se content in kale leaf was measured according to the method recommended by the Standardization Administration of the People’s Republic of China (GB 5009.93-2017) [24]. To determine the content, 0.5 g of kale leaves was acid-digested using 10 mL of HNO_3_ solution and 2 mL of H_2_O_2_ solution in a microwave digestion system (MARS6, CEM Corp., Matthews, NC, USA). Then, 5 mL of HCl solution (6 mol/L) was added and heated until it was clear and colorless, with white smoke emerging. After being cooled, the mixture was transferred to a 10 mL volumetric flask, where 2.5 mL of potassium ferricyanide solution was added and the remaining volume was made up of water. Finally, the solution was detected on an inductively coupled plasma mass spectrometer (iCAP RQ, Thermo Fisher Scientific, Waltham, MA, USA) using a mixed standard solution of 28 metal elements (including Se).

### 2.7. Determination of Speciation of Se Compounds in Kale Leaf

The speciation of Se compounds in kale leaf was detected using the method recommended by the Standardization Administration of the People’s Republic of China (NY/T 3556-2020) [25]. First, 1.0 g of kale leaves was placed in a 50 mL centrifuge tube, 10 mL of Tris-HCl buffer solution (30 mmol/L, pH 7.5) was added and the mixture underwent ultrasonication for 30 min. After the addition of 25 mg of protease (≥3.5 U/mg), the mixture was enzymatically hydrolyzed on a thermostatic oscillator (37 °C, 300 r/min) for 20 h. Then, the reaction mixture was taken out and centrifuged (5000 r/min, 10 min) to collect the supernatant. Subsequently, 2 mL of the supernatant was transferred to a 10 mL volumetric flask, and the remaining volume was made up of the mobile phase (15 mmol/L ammonium acetate, 0.2 mmol/L tetrabutyl ammonium hydroxide and 5% methanol; pH 5.5). After mixing, the samples were filtered using a 0.22 μm filter membrane and subjected to Se speciation determination on a high-performance liquid chromatography-inductively coupled plasma mass spectrometer (LC300-NexlON5000G, PerkinElmer, Waltham, MA, USA), using standard solutions of Se(IV), Se(VI), SeCys_2_, Se-MeSeCys and SeMet.

### 2.8. Quasi-Targeted Metabolomics Analysis of Kale Leaf

Kale leaves from the control group and the 10 mg/L Se group (labeled as the selenate group) were collected for quasi-targeted metabolomics analysis (each group had at least four replicates), which was performed according to the methods outlined in the study by Wu et al. [26], under the support of Novogene Co., Ltd. (Beijing, China). The procedures are provided in more detail below.

#### 2.8.1. Sample Preparation

Lyophilized kale leaves (100 mg) were placed in Eppendorf tubes, and 500 μL of a pre-cooled 80% methanol aqueous solution was added and extensively vortexed. The samples were kept standing in an ice bath for 5 min and then centrifuged (15,000× *g*, 20 min) at 4 °C to obtain the supernatant. Subsequently, some of the supernatant was diluted with LC-MS-grade water to a final concentration containing 53% methanol. The diluents underwent centrifugation (15,000× *g*, 20 min, 4 °C) to acquire supernatants and were then subjected to HPLC-MS/MS analysis.

#### 2.8.2. HPLC-MS/MS Analysis

HPLC-MS/MS analysis was performed using an ExionLC^TM^ AD system (SCIEX) coupled with a QTRAP^@^ 6500+ mass spectrometer (SCIEX) from Novogene Co., Ltd. (Beijing, China). Samples were eluted on an Xselect HSS T3 (2.1 × 150 mm, 2.5 μm) column (Waters, Milford, CT, USA) using eluent A (0.1% formic acid–water) and eluent B (0.1% formic acid–acetonitrile) at a flow rate of 0.4 mL/min, under the following gradient elution procedures: 0 min, 98% A and 2% B; 2 min, 98% A and 2% B; 15 min, 0% A and 100% B; 17 min, 0% A and 100% B; 17.1 min, 98% A and 2% B; and 20 min, 98% A and 2% B. Furthermore, the QTRAP^@^ 6500+ mass spectrometer was operated in a positive/negative polarity mode under the following conditions: curtain gas, 35 psi; collision gas, medium; ionspray voltage, 5500 V/−4500 V; temperature, 550 °C; ion source gas, 1:60; and ion source gas 2:60.

#### 2.8.3. Metabolite Identification and Quantification

The samples were detected using multiple reaction monitoring based on the Novogene database. Q1, Q3, RT (retention time), DP (declustering potential) and CE (collision energy) variables were used for the identification of metabolites, while Q3 alone was used for the quantification of metabolites. The data files acquired via HPLC-MS/MS analysis were opened using SCIEX OSV1.4 software, and then the integration and correction of the peaks were performed by this software. The main parameters for peak screening were as follows: minimum peak height, 500; signal/noise ratio, 5; and Gaussian smooth width, 1. The area of each peak represents the relative content of the corresponding metabolite.

#### 2.8.4. Data Analysis

The identified metabolites were annotated using the Kyoto Encyclopedia of Genes and Genomes (KEGG, https://www.genome.jp/kegg/pathway.html; accessed on 5 May 2024), Human Metabolome Database (HMDB, https://hmdb.ca/metabolites; accessed on 5 May 2024) and Lipidmaps (http://www.lipidmaps.org/; accessed on 5 May 2024) databases. Multivariate statistical analysis was conducted using principal component analysis (PCA) and partial least squares discriminant analysis (PLS-DA) on metaX 1.4.16 software, and univariate analysis was performed using Student’s *t*-test. The metabolites with variable importance in the projection (VIP) > 1, *p*-value < 0.05 and fold change ≥ 2 or fold change ≤ 0.5 were considered to be differential metabolites. Meanwhile, volcano plots drawn by ggplot2 in the R project were used to filter metabolites of interest, which were based on the VIP, Log2 (fold change) and −log10 (*p*-value) values. Moreover, KEGG (http://www.kegg.jp/; accessed on 5 May 2024) pathway analysis and KEGG enrichment analysis were carried out based on previously screened differential metabolites.

### 2.9. Correlation Analyses Between Metabolic Biomarkers, Se Contents and Se Speciation

Correlation analyses between metabolic biomarkers, Se contents and Se speciation were carried out by Pearson analysis in Origin 2022 software (OriginLab Corporation, Northampton, MA, USA), using the “Correlation Plot” package. The corresponding correlation heatmaps were processed and exported.

### 2.10. Statistical Analysis

All data are expressed as mean ± standard error. Differences between all experimental groups were examined by a one-way analysis of variance (ANOVA) combined with Tukey’s analysis, using SPSS 23.0 software (Chicago, IL, USA). In addition, a statistical analysis of the data from quasi-targeted metabolomics was performed using Student’s *t*-test. All results with *p*-values < 0.05 were considered statistically significant.

## 3. Results and Discussion

### 3.1. Effect of Se Fortification on the Growth Performance of Kale

**Figure 1:**
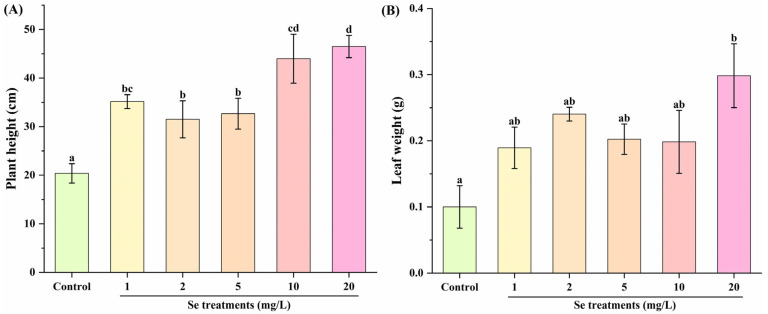
Effect of Se fortification on plant height (**A**) and leaf weight (**B**) of kale (*n* = 3). Different letters represent significant differences (*p* < 0.05) from each other.

Se fortification has been demonstrated to affect the growth performance of kale microgreens [14] and kale seedlings [12]. In Figure 1A, the plant height of kale in the control group was 20.39 ± 1.98 cm, while that in the Se treatment groups ranged from 31.50 ± 3.82 cm to 46.50 ± 2.29 cm. Compared with the control group, the plant height of kale was significantly increased in the Se treatment groups. No remarkable difference was seen in the plant height of kale between the 1, 2 and 5 mg/L Se groups, between the 1 and 10 mg/L Se groups and between the 10 and 20 mg/L Se groups. However, the plant height of kale in the 20 mg/L Se group was clearly higher than that in the 1, 2 and 5 mg/L Se groups. Meanwhile, in comparison with the 2 and 5 mg/L Se groups, the plant height of kale in the 10 mg/L Se group was dramatically higher. Similarly, the combined application of bio-slurry and inorganic nitrogen has been reported to markedly increase the plant height of kale [5]. As shown in Figure 1B, only the 20 mg/L Se treatment notably enhanced the leaf weight of kale (0.30 ± 0.05 g) compared to the control group (0.10 ± 0.03 g). However, compared with the control group, 1, 2, 5 and 10 mg/L Se treatments did not significantly influence the leaf weight of kale. Similarly, Se [12] and sulfur [2] supplementations elevated the weights of kale leaf and kale seedlings, respectively.

### 3.2. Effect of Se Fortification on Chlorophyll, Total Phenolic and Total Se Contents in Kale Leaf

**Figure 2:**
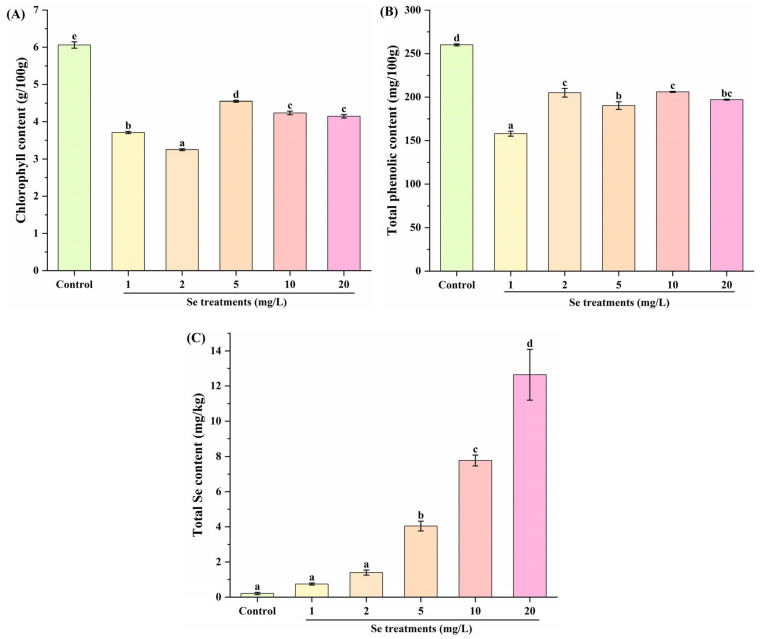
Effect of Se fortification on chlorophyll (**A**), total phenolic (**B**) and total Se contents (**C**) of kale leaf (*n* = 3). Different letters represent significant differences (*p* < 0.05) from each other.

The development and expansion of leaf were associated with an increase in chlorophyll content [27]. In Figure 2A, Se treatments (1, 2, 5, 10 and 20 mg/L) significantly decreased the chlorophyll content in kale leaf compared to the control group. Meanwhile, notable differences were observed among the 1, 2, 5, 10 and/or 20 mg/L Se groups, except between the 10 and 20 mg/L Se groups. In previous studies, no significant influence of Se on the accumulation of chlorophyll in kale microgreens was observed [28], whereas Se fortification notably added to the chlorophyll content in cauliflower [29]. Our observation revealed that Se treatments at 1, 2, 5, 10 and 20 mg/L could inhibit the biosynthesis of chlorophyll in kale leaf. This might be explained by the different species. Moreover, the results indicated that Se fortification might down-regulate the nutritional value of chlorophyll in kale leaf, which might in turn affect the metabolic profiles of other nutrients.

The accumulation of polyphenols in plants is usually associated with stress conditions during the growth cycle [30]. Moreover, phenolic compounds are important specialized metabolites that contribute to the health-promoting properties of kale [31]. In Figure 2B, Se treatments (1, 2, 5, 10 and 20 mg/L) also significantly decreased the total phenolic content in kale leaf, as compared with the control group. Similarly, Ortega-Hernández et al. [32] found that Se treatments markedly decreased the phenolic accumulation in kale sprouts. It might be speculated that Se treatments caused stress alterations to kale, which should be further confirmed as there were different findings in previous studies concerning other cultivars of kale or other plants. For example, in an investigation conducted by Viltres-Portales et al. [28], the total polyphenolic compound content of kale microgreens is not affected by Se treatment. And Se fortification has been demonstrated to significantly increase the concentration of some phenolic acids, especially chlorogenic and protocatechuic acids in kale sprouts [15]. At the same time, Se fortification notably elevated the total polyphenolic content in cauliflower [29]. Furthermore, compared with the 1 mg/L Se group, the total phenolic contents in kale leaf were observably increased in other Se treatment groups. Similarly, Paweł et al. [11] found that Se fortification stimulated the production of phenolic acids in kale sprouts, depending on the Se dose. Based on the above findings, it is necessary to determine the comprehensive changes in nutritional compounds of kale using metabolomics analysis.

One of the simplest and most robust techniques to increase the Se content in plants is by growing plants in high-Se soil and applying Se fertilizers [33]. As illustrated in Figure 2C, as compared to the control group, 5, 10 and 20 mg/L Se treatments notably up-regulated the total Se contents in kale leaf. Moreover, Se treatments significantly increased the total Se contents of kale leaf in a dose-dependent manner, in the Se concentration range of 2–20 mg/L. This was consistent with the previous findings that Se biofortification was effective in enriching the Se content in kale microgreens [14], seedlings [10,12] and sprouts [11,15].

### 3.3. Effect of Se Fortification on Speciation of Se Compounds in Kale Leaf

**Figure 3:**
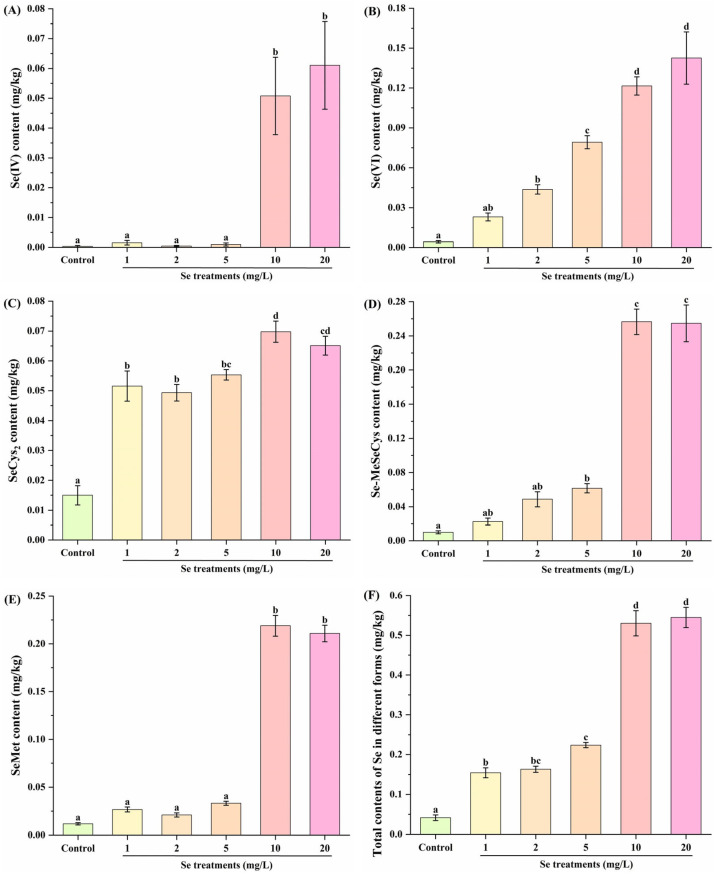
Effect of Se fortification on Se speciation in kale leaf (*n* = 3). (**A**) Se(IV) content; (**B**) Se(VI) content; (**C**) SeCys_2_ content; (**D**) Se-MeSeCys content; (**E**) SeMet content; (**F**) total contents of Se in different forms. Different letters represent significant differences (*p* < 0.05) from each other.

The speciation of Se in plant tissue is important for understanding the efficiency of Se absorption for animals and humans [34]. Both inorganic Se compounds (i.e., Se(IV) and Se(VI)) and organic Se compounds (i.e., SeCys_2_, Se-MeSeCys and SeMet) are frequently found in plants [35]. As shown in Figure 3A,E, the Se(IV) and SeMet contents in kale leaf were significantly increased in the 10 and 20 mg/L Se groups compared to those in the control group. In Figure 3C,F, Se fortifications (1–20 mg/L) notably elevated the SeCys_2_ content and total contents of Se in different forms in kale leaf.

In Figure 3B, compared with the control group, the Se(VI) content in kale leaf was clearly enhanced in the 2, 5, 10 and 20 mg/L Se groups. In Figure 3D, the Se-MeSeCys content in kale leaf was dramatically increased in the 5, 10 and 20 mg/L Se groups compared to that in the control group. These results indicate that Se fortification added inorganic and organic Se compounds to kale leaf. To the best of our knowledge, the median lethal doses of inorganic Se compounds (Se(IV) and Se(VI)) were 7 mg/kg·bw and ~13 mg/kg·bw, respectively. However, organic Se species can have greater bioactivities with less toxicity compared to inorganics species [33]. As a result, the bioactivities of kale leaf tend to be enriched by Se fortification. Furthermore, Figure 3 displays that the contents of organic Se compounds in Se-fortified kale are higher than those of inorganic Se compounds. From this perspective, Se fortification is desirable for kale as long as it is consumed within a safe dosage.

### 3.4. Effect of Se Fortification on Bioactive Compounds in Kale Leaf

**Figure 4:**
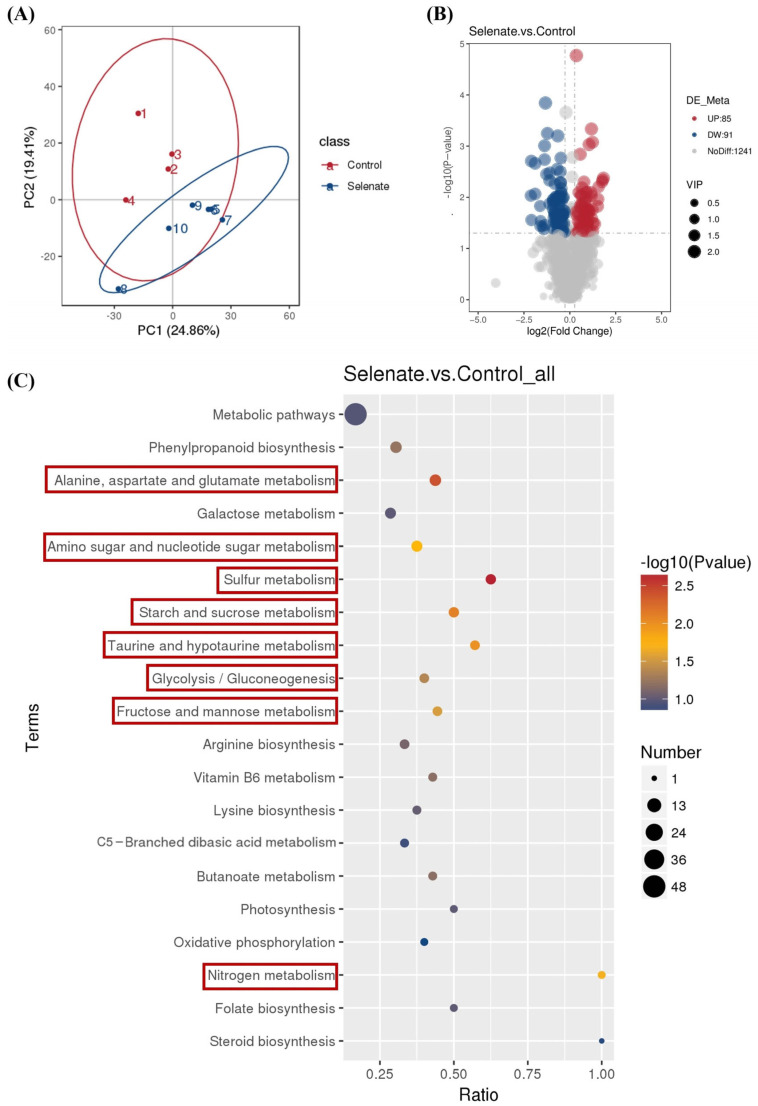
Effect of Se fortification on metabolic profile of kale leaf. (**A**) PCA score plot; (**B**) volcano plots of differential metabolites; (**C**) top 20 enriched KEGG pathways of differential metabolites.

There are few reports on the effect of selenium supplementation on the concentration of bioactive compounds present in kale plants [36]. Thus, quasi-targeted metabolomics analysis was performed to ascertain the effect of Se fortification on bioactive compounds in kale leaf. The representative total ion chromatograms of kale samples from the control and selenate groups in positive ion (ESI+) and negative ion (ESI−) modes can be seen in the Supplementary Materials (Figures S1 and S2). In the PCA score plot (Figure 4A), samples of kale leaf were clearly separated between the control group and the selenate group, suggesting that Se fortification changed the metabolic profile of kale leaf. As shown in Figure 4B, a total of 176 differential metabolites (85 up-regulated and 91 down-regulated) were identified. Among them, 113 and 63 differential metabolites were detected in positive ion (ESI+) and negative ion (ESI−) modes, respectively. The differential metabolites can be mainly classified into six types of compounds, including amino acid and its derivatives (Table 1); organic acid and its derivatives (Table 2); carbohydrates and its derivatives (Table 3); lipids (Table 4); flavonoids (Table 5); and organoheterocyclics (Table 6).

Using KEGG analysis based on these differential metabolites, 46 terms were enriched. The top 20 enriched KEGG pathways are displayed in Figure 4C. There were eight significantly enriched KEGG pathways, including alanine, aspartate and glutamate metabolism; amino sugar and nucleotide sugar metabolism; sulfur metabolism; starch and sucrose metabolism; taurine and hypotaurine metabolism; glycolysis/gluconeogenesis; fructose and mannose metabolism; and nitrogen metabolism. These metabolic pathways have been reported to be clearly affected by Se fortification or other treatments in kale or other plants. For example, high-voltage electrostatic field treatment has been demonstrated to significantly regulate alanine, aspartate and glutamate metabolism of kale [37]. Se fortification has been indicated to significantly regulate amino sugar and nucleotide sugar metabolism of alfalfa sprouts [38]. Plants absorb inorganic selenium and convert it into various organic selenides via the sulfur metabolism pathway [39]. Because the absorption and assimilation of sulfur by plants are coordinated with the absorption and assimilation of nitrogen, by influencing sulfur metabolism, selenium also affects nitrogen metabolism [40]. Se fortification has been demonstrated to facilitate the production of sulfur compounds in kale sprouts [15,41]. Se fortification has been found to markedly modulate starch and sucrose metabolism and glycolysis/gluconeogenesis metabolic pathways of alfalfa leaves [42]. Selenium nanoparticle treatment has been observed to clearly regulate taurine and hypotaurine metabolism in soybean seedlings [43]. Se treatment has been reported to dramatically regulate fructose and mannose metabolism in rice [44].

#### 3.4.1. Effect of Se Fortification on Amino Acid and Its Derivatives in Kale Leaf

After further analysis of the differential metabolites, 32 compounds (19 detected in ESI+ mode and 13 detected in ESI− mode) belonging to amino acid and its derivatives were identified, as shown in Table 1. Among them, 15 (i.e., trimethyllysine, 2-amino-2-deoxy-D-gluconate, homocysteine, L-asparagine, aspartate, ala-Gln, etc.) and 17 (D-threonine, L-glutamic acid, N alpha-acetyl-L-Arginine, ureidosuccinic acid, O-acetylserine, L-homoserine, etc.) compounds were significantly up-regulated and down-regulated, respectively. In particular, the quantitative values of trimethyllysine and ala-Gln in kale leaf were significantly increased to more than 2-fold in the selenate group compared to those in the control group. A previous study has reported that trimethyllysine is an important post-translationally modified amino acid with functions in the biosynthesis of carnitine, which is importantly involved in the transport of long-chain fatty acids from the cytosol to the mitochondria in both eukaryotes and some prokaryotes [45]. Moreover, Ala-Gln supplementation has been proven to possess an intestinal protection function [46,47]. On the other hand, the quantitative values of kynurenine, santacruzamate A and 3-(2-Naphthyl)-L-alanine clearly declined approximately 0.23-, 0.27- and 0.27-fold in the selenate group compared to those in the control group. Among them, 3-(2-Naphthyl)-L-alanine in dark tea has been proven to show significant correlations with digestive enzyme inhibition [48]. These observations indicated that Se fortification had significant influences on the contents of amino acid and its derivatives in kale leaf, which might enhance some biological activities like intestinal protection function.

#### 3.4.2. Effect of Se Fortification on Organic Acid and Its Derivatives in Kale Leaf

After further analysis of the differential metabolites, 19 compounds (9 identified in ESI+ mode and 10 detected in ESI− mode) belonging to organic acid and its derivatives were determined, as illustrated in Table 2. Of those, 9 compounds including N-Acetyl-L-carnosine, maleamic acid, 4-hydroxybenzoate, p-aminobenzoate, alpha-ketoglutaric acid, L-cysteinesulfinic acid, hydroxycitric acid and 2-aminoethanesulfonic acid were notably up-regulated, whereas 10 compounds including 2-aminoethylphosphonate, dodecanedioic acid, trans-aconitic acid, phosphoric acid, succinic acid, methylmalonate, glutaconic acid, 3-hydroxyanthranilic acid, vanillic acid and isovanillic acid were evidently down-regulated. In particular, the quantitative values of p-aminobenzoate and L-cysteinesulfinic acid were markedly enhanced to approximately 3.45- and 2.57-fold in the selenate group as compared to those in the control group. Of those, p-aminobenzoate has been reported to possess numerous biological activities, such as antioxidant, antibacterial, antimutagenic, anticoagulant, fibrinolytic and immunomodulating activities, protection against UV irradiation, as well as chemical induction associated with thermotolerance and pathogen resistance in plants [49]. L-Cysteinesulfinic acid has been demonstrated to have anti-methanogenic and antimicrobial activities [50]. These results suggested that Se fortification had significant effects on the contents of organic acid and its derivatives in kale leaf, which might elevate some biological activities like antibacterial action.

#### 3.4.3. Effect of Se Fortification on Carbohydrates and Their Derivatives in Kale Leaf

After further analysis of the differential metabolites, 25 compounds (7 detected in ESI+ mode and 18 detected in ESI− mode) belonging to carbohydrates and their derivatives were identified, as displayed in Table 3. Of those, 22 (i.e., isomaltose, DIMBOA glucoside, icariside B2, sorbitol-6-phosphate, D-glucose 1-phosphate, D-fructose 6-phosphate, etc.) and 3 (D-threose, pteroside A and sedoheptulose anhydride) compounds were dramatically up-regulated and down-regulated, respectively. In particular, the quantitative value of DIMBOA glucoside in kale leaf was significantly increased to be approximately 2.89-fold in the selenate group compared to that in the control group. It is worth noting that DIMBOA glucoside has been reported to enhance the resistance of wheat and maize to aphids [51]. Moreover, low-molecular-weight carbohydrates are compounds directly related to the kale flavor and nutritional quality [52]. According to Table 3, the quantitative values of D-glucopyranose, D-galactaric acid, L-sorbose, D-glucuronic acid, D-galactose, alpha-D-glucose, D-ribose, D-glucose and D-xylose were notably up-regulated. Among them, glucose is one of the major soluble sugars found in kale [3,4]. These findings implied that Se fortification had notable impacts on the amounts of carbohydrates and their derivatives in kale leaf, which might alter the kale flavor and nutritional quality.

#### 3.4.4. Effect of Se Fortification on Lipids in Kale Leaf

Plant lipids have become increasingly popular as functional components in the creation of functional foods among all bioactive phytochemicals [3]. After further analysis of the differential metabolites, 17 compounds (12 identified in ESI+ mode and 5 identified in ESI− mode) belonging to lipids were determined, as revealed in Table 4. Among them, 4 (methyl oleate, 1-hexadecanol, (E)-3-(4-Hydroxyphenyl)propenoic acid (2S)-3-(beta-D-glucopyranosyloxy)-2-hydroxypropyl ester and hexadecanedioic acid) and 13 (i.e., palmitoleic acid, LPC (1-acyl 16:2), trans-2-hexenal, LysoPC 18:3 (2n isomer), 9,10-EODE, 3-methyladipic acid, etc.) compounds were significantly up-regulated and down-regulated, respectively. In particular, the quantitative value of 1-hexadecanol in kale leaf was observably increased to be more than 2-fold in the selenate group compared to that in the control group. In previous studies, 1-hexadecanol has been found to exhibit antimicrobial [53] and anticancer [54] activities. These results hinted that Se fortification produced clear influences on the contents of lipids in kale leaf, which might enhance some bioactivities.

#### 3.4.5. Effect of Se Fortification on Flavonoids in Kale Leaf

Flavonoids, being prevalent in vegetables, are essential to the diverse stages of their growth, development and storage [55], and they have been shown to have multiple pharmacological activities, such as antioxidant, anti-inflammatory, antibacterial, antiviral, anticancer, antidiabetic and immune function modulation [56]. Kale is a good source of flavonoids compared to other commonly consumed vegetables [4]. Organic and bio-organic fertilizers have been demonstrated to enhance the flavonoids of broccoli (*Brassica leracea*, Var. *Italica*) [57]. After further analysis of the differential metabolites, 14 compounds (10 detected in ESI+ mode and 4 detected in ESI− mode) belonging to flavonoids were identified, as listed in Table 5. Of those, seven compounds (8-C-hexosyl-apigenin O-hexosyl-O-hexoside, methylChrysoeriol-5-O-hexoside, irisolidone-7-O-beta-d-glucoside, quercetin-O-glucoside, C-pentosyl-chrysoeriol 7-O-feruloylhexoside, chrysin O-malonylhexoside and sakuranetin) were notably up-regulated, and seven compounds (methylLuteolin-C-hexoside, daidzin, chrysin O-hexoside, 4′-O-glucosylvitexin, genistin, heptamethoxyflavone and azaleatin) were notably down-regulated. In particular, the quantitative value of C-pentosyl-chrysoeriol 7-O-feruloylhexoside in kale leaf was significantly increased to approximately 2.86-fold in the selenate group compared to that in the control group. Similarly, the C-pentosyl-chrysoeriol 7-O-feruloylhexoside content of goji fruit was significantly increased with phosphorus fertilizer levels [58]. These observations indicated that Se fortification generated significant effects on the contents of flavonoids in kale leaf, which might elevate some pharmacological activities.

#### 3.4.6. Effect of Se Fortification on Organoheterocyclic Compounds in Kale Leaf

Organoheterocyclic compounds have been reported for the treatment of cancer and kidney diseases [59]. Moreover, the accumulation of glucosinolates induced by thermal stress has been found to significantly affect organoheterocyclic compounds of kale [60]. After further analysis of the differential metabolites, 14 compounds (12 identified in ESI+ mode and 2 identified in ESI− mode) belonging to organoheterocyclic compounds were determined, as shown in Table 6. Among them, 4 compounds (deoxynojirimycin, 3-indoleacetonitrile, NNK and indole-2-carboxylic acid) were significantly up-regulated, and 10 compounds (i.e., hypoxanthine, zarzissine, N-methylnicotinamide, (S)-2-phenyloxirane, 4-pyridoxate, brazilin, etc.) were significantly down-regulated, respectively. In particular, the quantitative value of indole-2-carboxylic acid was markedly up-regulated to approximately 2.19-fold in the selenate group as compared to that in the control group. In a previous report [61], indole-2-carboxylic acids have been demonstrated to be MCL-1 inhibitors, thereby showed anticancer activity. These findings suggested that Se fortification had significant impacts on the amounts of organoheterocyclic compounds in kale leaf, which might alter some bioactivities.

#### 3.4.7. Effect of Se Fortification on Other Differential Metabolites in Kale Leaf

There were other differential metabolites screened between the selenate group and the control group, including 10 phenylpropanoids and polyketides, 12 nucleotides and their derivates, 6 terpenoids, 4 amines, 5 vitamins, 4 phenols and their derivatives, 6 phenolic acids, 2 phytohormones, 2 alkaloids and their derivatives, 1 polyamine, 1 organooxygen compound, 1 benzene and substituted derivatives and 1 alcohol and polyol, as shown in Table 7. In a previous study [62], terpenoids and alkaloids were speculated to be bioactive constituents for the antibacterial activity of kale leaf extracts. Moreover, Se fortification has been widely reported to affect the synthesis of phenolic compounds in kale sprouts, which is related to cytotoxic, antioxidant and anti-inflammatory activities [11,15,32,41]. Furthermore, phytohormones are known to participate in plant Se accumulation [63], and polyamines are involved in the regulation of the cellular growth, apoptosis, rooting, flower development and stress responses of plants [64]. In Table 7, the quantitative value of caffeic acid in kale leaf is significantly increased to more than 2-fold in the selenate group compared to that in the control group. It has been reported that caffeic acid is one of the most abundant phenolic acids in kale leaves, and this compound is highly correlated with antioxidant and antibacterial activities [65]. These results implied that Se fortification produced significant effects on the contents of other differential metabolites like phenylpropanoids and polyketides in kale leaf, which might enhance some bioactivities.

### 3.5. Metabolic Biomarkers in Kale Leaf Affected by Se Fortification

**Figure 5:**
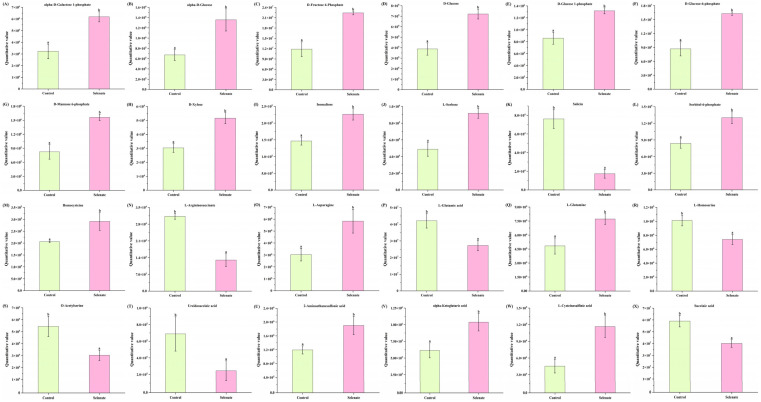
The levels of the 24 metabolic biomarkers (enriched in 8 metabolic pathways of alanine, aspartate and glutamate metabolism, amino sugar and nucleotide sugar metabolism, sulfur metabolism, starch and sucrose metabolism, taurine and hypotaurine metabolism, glycolysis/gluconeogenesis, fructose and mannose metabolism and nitrogen metabolism) in kale leaf of selenate group vs. control group. Different letters (a and b) represent significant differences (*p* < 0.05) from each other.

As mentioned in Figure 4, eight different metabolic pathways of kale leaf that were significantly regulated by Se fortification were determined. When combined, there were 24 differential metabolites (12 carbohydrates and their derivatives, 8 amino acids and their derivatives and 4 organic acid and their derivatives) enriched in the eight metabolic pathways, as shown in Figure 5. These 24 metabolites could be considered as biomarkers indicating the changes in nutrients of kale caused by selenium fortification. The interaction of sugars and amino acids could affect plant quality and aroma [66]. Moreover, the contents of amino acids could influence nitrogen accumulation in plants [67], and amino acids’ or compounds’ synthesis play important roles in the adaptions of plants to stress and adversity [68]. In Figure 5, the quantitative values of 17 (i.e., alpha-D-galactose 1-phosphate, alpha-D-glucose, D-fructose 6-phosphate, D-glucose, homocysteine and L-asparagine, etc.) and 7 (salicin, L-argininosuccinate, L-glutamic acid, L-homoserine, O-acetylserine, ureidosuccinic acid and succinic acid) compounds ae notably up-regulated and down-regulated, respectively. Similarly, nano-Se foliar application increased the glucose content in *Siraitia grosvenorii* [66]. The D-glucose-6-phosphate content was significantly increased, whilst that of L-glutamic acid was dramatically decreased in alfalfa affected by Se fortification [67]. L-Asparagine and L-glutamine in red pitaya fruit have been demonstrated to be greatly increased by nano-selenium biofortification [68]. Of note, some of the above-metabolic biomarkers have been demonstrated to possess health benefits. For instance, 2-aminoethanesulfonic acid (also known as taurine) has been reported to play important roles in the treatment of diseases of the muscle, the central nervous system and the cardiovascular system [69]. Alpha-ketoglutaric acid is involved in both energy metabolism and carbon and nitrogen metabolism, exhibiting a variety of functions, and therefore it may act as a regulator in the progression of a variety of diseases [70]. L-Cysteinesulfinic acid has been proven to modulate cardiovascular function in the periaqueductal gray area of rats [71]. Therefore, the changes in metabolic biomarkers induced by Se fortification might be related to the growth, quality and nutritive value of kale. This speculation should be confirmed in the future.

### 3.6. Correlations Between Metabolic Biomarkers and Se Contents as Well as Speciation

**Figure 6:**
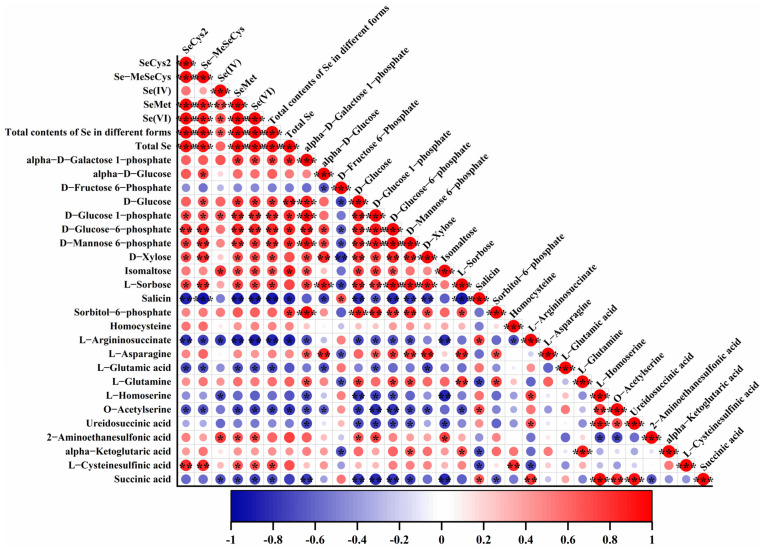
Pearson correlation analysis between the 24 metabolic biomarkers and Se contents as well as speciation in kale. *, ** and *** indicate a significant difference value of 0.05, 0.01 and 0.001, respectively.

Pearson correlation analyses were performed to explore whether the contents of metabolic biomarkers in kale leaf were related to Se contents and speciation, as illustrated in Figure 6. Many significant positive correlations were observed between carbohydrates and their derivatives and Se contents as well as speciation: SeCys_2_ with D-glucose 1-phosphate, D-glucose-6-phosphate, D-mannose 6-phosphate, D-xylose and L-sorbose; Se-MeSeCys with alpha-D-glucose, D-glucose, D-glucose 1-phosphate, D-glucose-6-phosphate, D-mannose 6-phosphate, D-xylose and L-sorbose; Se(IV) with D-glucose 1-phosphate and isomaltose; SeMet and Se(VI) with alpha-D-galactose 1-phosphate, D-glucose, D-glucose 1-phosphate, D-glucose-6-phosphate, D-mannose 6-phosphate, D-xylose, isomaltose, L-sorbose; total contents of Se in different forms with alpha-D-galactose 1-phosphate, D-glucose, D-glucose 1-phosphate, D-glucose-6-phosphate, D-mannose 6-phosphate, D-xylose, isomaltose, L-sorbose; total Se with alpha-D-galactose 1-phosphate, D-glucose, D-glucose 1-phosphate, D-glucose-6-phosphate, D-mannose 6-phosphate, isomaltose and sorbitol-6-phosphate. Meanwhile, some clear positive correlations were seen between organic acid and its derivatives and Se contents as well as speciation: SeCys_2_ with L-cysteinesulfinic acid; Se-MeSeCys with L-cysteinesulfinic acid; Se(IV) with 2-aminoethanesulfonic acid; SeMet and Se(VI) with 2-aminoethanesulfonic acid and L-cysteinesulfinic acid; total contents of Se in different forms with L-cysteinesulfinic acid.

On the other hand, some remarkable negative correlations were seen between amino acid and its derivatives and Se contents as well as speciation: SeCys_2_ and Se-MeSeCys with L-argininosuccinate, L-glutamic acid and O-acetylserine; Se(IV) with L-argininosuccinate and L-homoserine; SeMet, Se(VI) and total contents of Se in different forms with L-argininosuccinate, L-glutamic acid and O-acetylserine; total Se with L-argininosuccinate and O-acetylserine. Moreover, SeCys_2_, Se-MeSeCys, Se(IV), SeMet, Se(VI), total contents of Se in different forms and total Se showed significant negative correlations with one (salicin) of the carbohydrates and its derivatives. Meanwhile, Se(IV), SeMet, Se(VI) and total contents of Se in different forms were clearly negatively correlated with one (succinic acid) organic acid and its derivatives.

Furthermore, prominent positive and negative correlations were found in themselves of metabolic biomarkers or Se contents as well as speciation. Similarly, clear correlations between differential metabolites and Se along with differential metabolites and differential metabolites have been discovered in purple rice grains exposed to different selenium concentrations [72]. In the study by Zagrodzki et al. [11], Se was proven to be correlated only with caffeic acid in Se fortification of kale. Paśko et al. [15] found that Se speciation in kale sprouts fortified with novel organic Se compounds was strongly correlated with one another.

In general, Se fortification produced positive effects on the accumulation of carbohydrates and their derivatives along with organic acid and its derivatives, whereas this generated negative influences on the production of amino acid and its derivatives.

## 4. Conclusions

Se deficiency is an urgent problem that needs to be addressed, and Se fortification is commonly applied to produce Se-enriched foods. In the present study, Se fortification using Se fertilizer significantly increased the contents of total Se and different Se speciation in kale leaf. Meanwhile, Se fortification clearly increased the plant height and leaf weight of kale, and it notably decreased the chlorophyll and total phenolic contents in kale leaf. Moreover, Se fortification markedly changed the metabolic profile of kale leaf, especially involved in six types of compounds and eight metabolic pathways. Twenty-four metabolites were screened as biomarkers indicating the changes in nutrients of kale caused by selenium fortification. Furthermore, correlation analysis reflected that Se fortification produced positive effects on the accumulation of carbohydrates and their derivatives as well as organic acid and its derivatives, while it generated negative influences on the production of amino acid and its derivatives, in kale leaf. These results indicate that Se fortification has a significant influence on growth performance and nutritional compounds of kale. In contrast with previous studies, our findings are beneficial for comprehensively understanding the impact of selenium fortification on the nutritional compounds in kale, and they can guide future research on the potential biological activities of Se-biofortified kale. This is of great significance for the development and future research of the kale industry. However, several limitations of this study should be addressed in future research: the nutritive value and bioactivity of Se-enriched kale remain to be thoroughly investigated; the mechanisms through which selenium fortification influences the growth performance and nutritional compounds of kale require further elucidation; the effects of other fortification methods on kale growth and nutrients of kale also merit comparative study. In the next work, we will focus on resolving these issues.

## Figures and Tables

**Table 1:** Significantly differential metabolites belonging to amino acid and its derivatives in selenate group vs. control group.

Number	RT (min)	Name	Formula	Molecular Weight (Da)	Fold Change	*p*-Value	Change
**ESI+ mode**							
1	0.760	Trimethyllysine	C_9_H_21_N_2_O_2_	189.275	2.96414	0.006252	Up
2	0.840	2-Amino-2-deoxy-D-gluconate	C_6_H_13_NO_6_	195.171	1.49011	0.016303	Up
3	0.840	Homocysteine	C_4_H_9_NO_2_S	135.185	1.41606	0.033335	Up
4	0.860	D-Threonine	C_4_H_9_NO_3_	119.119	0.69360	0.048622	Down
5	0.880	N-Hydroxyl-tryptamine	C_10_H_12_N_2_O	176.215	1.64435	0.045438	Up
6	0.886	L-Glutamic acid	C_5_H_9_NO_4_	147.130	0.64700	0.025971	Down
7	0.960	N-Acetyl-L-valine	C_7_H_13_NO_3_	159.183	1.58498	0.006555	Up
8	1.000	D-Proline betaine	C_7_H_13_NO_2_	143.180	1.30347	0.022577	Up
9	1.008	Stachydrine	C_7_H_13_NO_2_	143.180	1.82899	0.008002	Up
10	1.040	S-Methyl-L-cysteine	C_4_H_9_NO_2_S	135.185	1.28090	1.70E-05	Up
11	1.040	N alpha-Acetyl-L-arginine	C_8_H_16_N_4_O_3_	216.238	0.69416	0.024535	Down
12	1.830	Homocysteic acid	C_4_H_9_NO_5_S	183.180	0.79828	0.035047	Down
13	1.840	L-Methionine sulfone	C_5_H_11_NO_4_S	181.210	0.77200	0.010351	Down
14	1.840	N-Acetyl-Dl-glutamic acid	C_7_H_11_NO_5_	189.166	1.39598	0.019346	Up
15	1.860	D-Homocysteine	C_4_H_9_NO_2_S	135.185	0.73808	0.011723	Down
16	2.270	DL-m-Tyrosine	C_9_H_11_NO_3_	181.190	0.69750	0.005192	Down
17	3.780	Kynurenine	C_10_H_12_N_2_O_3_	208.214	0.23397	0.027744	Down
18	8.680	Santacruzamate A	C_15_H_22_N_2_O_3_	278.350	0.26958	0.002170	Down
19	18.290	N-Acetyltryptamine	C_12_H_14_N_2_O	202.252	1.24839	0.049374	Up
**ESI− mode**							
1	0.843	L-Asparagine	C_4_H_8_N_2_O_3_	132.120	1.92649	0.030460	Up
2	0.850	Aspartate	C_4_H_7_NO_4_	133.103	1.46312	0.014558	Up
3	0.850	Ala-Gln	C_8_H_15_N_3_O_4_	217.220	2.42031	0.044904	Up
4	0.868	L-Glutamine	C_5_H_10_N_2_O_3_	146.140	1.59856	0.045253	Up
5	1.031	Ureidosuccinic acid	C_5_H_8_N_2_O_5_	176.130	0.35666	0.037044	Down
6	1.056	O-Acetylserine	C_5_H_9_NO_4_	147.130	0.55573	0.020191	Down
7	1.110	Allysine	C_6_H_11_NO_3_	145.074	1.65456	0.017013	Up
8	1.900	L-Homoserine	C_4_H_9_NO_3_	119.119	0.73223	0.035539	Down
9	2.150	N-Acetylalanine	C_5_H_9_NO_3_	131.130	0.60878	0.029064	Down
10	4.090	Phenprobamate	C_10_H_13_NO_2_	179.216	0.74104	0.025886	Down
11	5.410	L-Argininosuccinate	C_10_H_18_N_4_O_6_	290.273	0.41486	0.003546	Down
12	6.070	3-(2-Naphthyl)-L-alanine	C_13_H_13_NO_2_	215.095	0.26935	0.008577	Down
13	6.220	3-(2-Naphthyl)-D-alanine	C_13_H_13_NO_2_	215.248	0.76105	0.021510	Down

**Table 2:** Significantly differential metabolites belonging to organic acid and its derivatives in selenate group vs. control group.

Number	RT (min)	Name	Formula	Molecular Weight (Da)	Fold Change	*p*-Value	Change
**ESI+ mode**							
1	0.980	N-Acetyl-L-carnosine	C_11_H_16_N_4_O_4_	268.270	1.39197	0.008014	Up
2	1.490	Maleamic acid	C_4_H_5_NO_3_	115.090	2.00340	0.047445	Up
3	4.120	4-Hydroxybenzoate	C_7_H_6_O_3_	138.121	1.27311	0.018775	Up
4	4.450	p-Aminobenzoate	C_7_H_7_NO_2_	137.136	3.45323	0.004585	Up
5	6.070	4-Hydroxy-3,5-diisopropylbenzaldehyde	C_13_H_18_O_2_	206.200	1.69562	0.029888	Up
6	6.470	2-Aminoethylphosphonate	C_2_H_8_NO_3_P	125.064	0.56516	0.021502	Down
7	9.452	Dodecanedioic acid	C_12_H_22_O_4_	230.300	0.32932	0.036416	Down
8	10.950	trans-Aconitic acid	C_6_H_6_O_6_	174.016	0.69121	0.009376	Down
9	11.010	Phosphoric acid	H_3_O_4_P	97.995	0.79450	0.018272	Down
**ESI** **− mode**							
1	0.870	alpha-Ketoglutaric acid	C_5_H_6_O_5_	146.098	1.68713	0.041975	Up
2	0.880	L-Cysteinesulfinic acid	C_3_H_7_NO_4_S	153.160	2.57192	0.033105	Up
3	0.900	Hydroxycitric acid	C_6_H_8_O_8_	208.124	1.44818	0.037908	Up
4	1.040	2-Aminoethanesulfonic acid	C_2_H_7_NO_3_S	125.015	1.59222	0.045750	Up
5	1.936	Succinic acid	C_4_H_6_O_4_	118.090	0.67941	0.011762	Down
6	1.938	Methylmalonate	C_4_H_6_O_4_	118.090	0.66665	0.013200	Down
7	3.420	Glutaconic acid	C_5_H_6_O_4_	130.099	0.64917	0.009588	Down
8	4.450	3-Hydroxyanthranilic acid	C_7_H_7_NO_3_	153.135	0.58887	0.031454	Down
9	5.901	Vanillic acid	C_8_H_8_O_4_	168.150	0.53096	0.011337	Down
10	6.100	Isovanillic acid	C_8_H8O_4_	168.150	0.55737	0.012052	Down

**Table 3:** Significantly differential metabolites belonging to carbohydrates and their derivatives in selenate group vs. control group.

Number	RT (min)	Name	Formula	Molecular Weight (Da)	Fold Change	*p*-Value	Change
**ESI+ mode**							
1	0.900	Isomaltose	C_12_H_22_O_11_	342.297	1.54350	0.008773	Up
2	1.660	D-Threose	C_4_H_8_O_4_	120.042	0.66552	0.024747	Down
3	4.260	DIMBOA glucoside	C_15_H_19_NO_10_	374.108	2.88963	0.010864	Up
4	5.730	Pteroside A	C_21_H_30_O_8_	410.464	0.74440	0.045354	Down
5	6.050	Icariside B2	C_19_H_30_O_8_	386.194	1.95132	0.007890	Up
6	7.470	7-O-Methylaloeresin A	C_29_H_30_O_11_	554.550	1.96790	0.022436	Up
7	10.150	Sedoheptulose anhydride	C_7_H_12_O_6_	192.167	0.79070	0.009938	Down
**ESI** **− mode**							
1	0.860	Sorbitol-6-phosphate	C_6_H_15_O_9_P	262.045	1.55425	0.013097	Up
2	0.860	D-Glucose 1-phosphate	C_6_H_13_O_9_P	260.136	1.53388	0.022855	Up
3	0.860	D-Fructose 6-phosphate	C_6_H_13_O_9_P	260.136	1.89067	0.033529	Up
4	0.860	D-Glucose-6-phosphate	C_6_H_13_O_9_P	260.030	1.87176	0.036772	Up
5	0.870	D-Glucopyranose	C_6_H_12_O_6_	180.156	1.73418	0.019685	Up
6	0.870	D-Galactaric acid	C_6_H_10_O_8_	210.038	1.44618	0.021811	Up
7	0.870	alpha-D-Galactose 1-phosphate	C_6_H_13_O_9_P	260.136	1.91270	0.032810	Up
8	0.870	D-Mannose 6-phosphate	C_6_H_13_O_9_P	260.136	1.89630	0.044741	Up
9	0.890	L-Sorbose	C_6_H_12_O_6_	180.160	1.88091	0.033118	Up
10	0.894	D-Glucuronic acid	C_6_H_10_O_7_	194.140	1.88042	0.046651	Up
11	0.900	D-Galactose	C_6_H_12_O_6_	180.160	1.82545	0.033758	Up
12	0.910	6-Phosphogluconic acid	C_6_H_13_O_10_P	276.135	1.64561	0.039733	Up
13	0.912	alpha-D-Glucose	C_6_H_12_O_6_	180.160	2.01494	0.027591	Up
14	0.920	D-Ribose	C_5_H_10_O_5_	150.130	1.48251	0.001443	Up
15	0.940	D-Glucose	C_6_H_12_O_6_	180.156	1.85706	0.031938	Up
16	0.984	N-Acetylglucosamine	C_8_H_15_NO_6_	221.210	1.54461	0.009389	Up
17	1.010	D-Xylose	C_5_H_10_O_5_	150.131	1.70502	0.005010	Up
18	4.989	Salicin	C_13_H_18_O_7_	286.280	0.22890	0.009265	Down

**Table 4:** Significantly differential metabolites belonging to lipids in selenate group vs. control group.

Number	RT (min)	Name	Formula	Molecular Weight (Da)	Fold Change	*p*-Value	Change
**ESI+ mode**							
1	1.060	Methyl oleate	C_19_H_36_O_2_	296.488	1.81502	0.039804	Up
2	5.250	1-Hexadecanol	C_16_H_34_O	242.441	3.55889	0.004228	Up
3	10.900	Palmitoleic acid	C_16_H_30_O_2_	254.408	0.79256	0.032195	Down
4	10.990	LPC (1-acyl 16:2)	C_24_H_46_NO_7_P	491.600	0.55850	0.019798	Down
5	11.005	trans-2-Hexenal	C_6_H_10_O	98.140	0.76749	0.022241	Down
6	11.330	LysoPC 18:3 (2n isomer)	C_26_H_48_NO_7_P	517.200	0.69023	0.026496	Down
7	11.360	LysoPC 14:0 (2n isomer)	C_22_H_46_NO_7_P	467.300	0.38479	0.000983	Down
8	11.370	Lysopc 14:0	C_22_H_46_NO_7_P	467.577	0.38528	0.001830	Down
9	11.450	LysoPC 15:1	C_23_H_46_NO_7_P	479.100	0.59558	0.026791	Down
10	11.540	LPC(1-acyl 16:1)	C_24_H_48_NO_7_P	493.610	0.62069	0.002815	Down
11	11.570	LysoPC 16:1	C_24_H_48_NO_7_P	493.300	0.64477	0.009996	Down
12	11.940	LysoPC 15:0	C_23_H_48_NO_7_P	481.300	0.78497	0.044473	Down
**ESI** **− mode**							
1	1.690	9,10-EODE	C_18_H_32_O_3_	296.000	0.61987	0.040417	Down
2	1.860	(E)-3-(4-Hydroxyphenyl)Propenoic Acid (2S)-3-(beta-D-Glucopyranosyloxy)-2-Hydroxypropyl Ester	C_18_H_24_O_10_	400.377	2.32277	0.025892	Up
3	5.747	3-Methyladipic acid	C_7_H_12_O_4_	160.170	0.74442	0.034135	Down
4	11.270	Lysope 14:0	C_19_H_40_NO_7_P	425.500	0.42945	0.000565	Down
5	11.908	Hexadecanedioic acid	C_16_H_30_O_4_	286.410	1.44060	0.040142	Up

**Table 5:** Significantly differential metabolites belonging to flavonoids in selenate group vs. control group.

Number	RT (min)	Name	Formula	Molecular Weight (Da)	Fold Change	*p*-Value	Change
**ESI+ mode**							
1	0.710	methylLuteolin-C-hexoside	C_22_H_22_O_11_	448.400	0.39236	0.034781	Down
2	0.900	8-C-hexosyl-apigenin O-hexosyl-O-hexoside	C_33_H_40_O_20_	756.669	1.97583	0.017199	Up
3	0.970	methylChrysoeriol-5-O-hexoside	C_23_H_24_O_11_	476.430	2.06676	0.000935	Up
4	0.970	Irisolidone-7-O-beta-d-glucoside	C_23_H_24_O_11_	476.430	1.59313	0.013537	Up
5	6.180	Daidzin	C_21_H_20_O_9_	416.111	0.52885	0.016847	Down
6	6.190	Chrysin O-hexoside	C_21_H_20_O_9_	416.200	0.46892	0.003323	Down
7	6.280	4′-O-Glucosylvitexin	C_27_H_30_O_15_	594.518	0.66480	0.042288	Down
8	6.540	Quercetin-O-glucoside	C_21_H_20_O_12_	464.376	2.10679	0.018089	Up
9	7.520	C-pentosyl-chrysoeriol 7-O-feruloylhexoside	C_37_H_38_O_18_	770.210	2.85714	0.014988	Up
10	7.590	Chrysin O-malonylhexoside	C_24_H_22_O_12_	502.431	2.10725	0.019679	Up
**ESI− mode**							
1	1.010	Sakuranetin	C_16_H_14_O_5_	286.279	1.72006	0.020090	Up
2	6.780	Genistin	C_21_H_20_O_10_	432.380	0.53560	0.010864	Down
3	7.080	Heptamethoxyflavone	C_22_H_24_O_9_	432.420	0.65885	0.011248	Down
4	7.300	Azaleatin	C_16_H_12_O_7_	316.265	0.37452	0.041797	Down

**Table 6:** Significantly differential metabolites belonging to organoheterocyclic compounds in selenate group vs. control group.

Number	RT (min)	Name	Formula	Molecular Weight (Da)	Fold Change	*p*-Value	Change
**ESI+ mode**							
1	0.730	Deoxynojirimycin	C_6_H_13_NO_4_	163.173	1.63225	0.044442	Up
2	1.090	3-Indoleacetonitrile	C_10_H_8_N_2_	156.180	1.74573	0.028890	Up
3	1.509	Hypoxanthine	C_5_H_4_N_4_O	136.110	0.58954	0.009671	Down
4	1.830	Zarzissine	C_5_H_5_N_5_	135.054	0.71998	0.008105	Down
5	1.840	N-Methylnicotinamide	C_7_H_8_N_2_O	136.151	0.72667	0.006758	Down
6	2.010	(S)-2-Phenyloxirane	C_8_H_8_O	120.058	0.74614	0.032044	Down
7	2.910	4-Pyridoxate	C_8_H_9_NO_4_	183.161	0.23329	0.001957	Down
8	3.950	4-Methyl-5-thiazoleethanol	C_6_H_9_NOS	143.100	0.54310	0.030762	Down
9	4.480	N-gamma-Acetyl-N-2-Formyl-5-Methoxykynurenamine	C_13_H_16_N_2_O_4_	264.277	0.54189	0.013823	Down
10	5.898	NNK	C_10_H_13_N_3_O_2_	207.230	1.87094	0.028485	Up
11	7.142	Indole-3-carboxylic acid	C_9_H_7_NO_2_	161.160	0.46336	0.004700	Down
12	12.565	Alantolactone	C_15_H_20_O_2_	232.320	0.63620	0.042875	Down
**ESI− mode**							
1	0.927	Indole-2-carboxylic acid	C_9_H_7_NO_2_	161.160	2.19170	0.045829	Up
2	5.905	Brazilin	C_16_H_14_O_5_	286.279	0.53742	0.025127	Down

**Table 7:** Other differential metabolites in selenate group vs. control group.

Number	RT (min)	Name	Formula	Molecular Weight (Da)	Class	Fold Change	*p*-Value	Change
**ESI+ mode**								
1	0.890	4-Hydroxycinnamaldehyde	C_9_H_8_O_2_	148.159	Phenylpropanoids and polyketides	0.68576	0.034948	Down
2	1.840	Caffeic aldehyde	C_9_H_8_O_3_	164.100	Phenylpropanoids and polyketides	0.70968	0.005165	Down
3	5.720	4-Methylumbelliferyl phenylphosphonate	C_16_H_13_O_5_P	316.240	Phenylpropanoids and polyketides	1.83270	0.048113	Up
4	6.424	Fangchinoline	C_37_H_40_N_2_O_6_	608.720	Phenylpropanoids and polyketides	0.39810	0.000144	Down
5	7.158	Dihydrocoumarin	C_9_H_8_O_2_	148.159	Phenylpropanoids and polyketides	0.81987	0.018214	Down
6	9.238	6-Methylcoumarin	C_10_H_8_O_2_	160.170	Phenylpropanoids and polyketides	1.84664	0.010285	Up
7	9.290	Ethyl coumarin-3-carboxylate	C_12_H_10_O_4_	218.205	Phenylpropanoids and polyketides	0.58299	0.023577	Down
8	10.992	Methyl eugenol	C_11_H_14_O_2_	178.230	Phenylpropanoids and polyketides	0.63596	0.000633	Down
9	0.960	2′-deoxy-5-(hydroxymethyl)cytidine	C_10_H_15_N_3_O_5_	257.240	Nucleotide and its derivates	1.34418	0.048231	Up
10	1.029	Cytidine	C_9_H_13_N_3_O_5_	243.220	Nucleotide and its derivates	1.74257	0.034440	Up
11	1.030	NADP	C_21_H_28_N_7_O_17_P_3_	743.400	Nucleotide and its derivates	0.32475	0.014813	Down
12	3.928	Xanthosine	C_10_H_12_N_4_O_6_	284.225	Nucleotide and its derivates	0.46649	0.013369	Down
13	4.040	1-Methylguanosine	C_11_H_15_N_5_O_5_	297.267	Nucleotide and its derivates	0.64768	0.023146	Down
14	4.140	2′-O-methyladenosine	C_11_H_15_N_5_O_4_	281.268	Nucleotide and its derivates	1.96054	0.017207	Up
15	4.540	N6-Succinyl adenosine	C_14_H_17_N_5_O_8_	383.313	Nucleotide and its derivates	0.71137	0.001703	Down
16	5.130	3′-Aenylic acid	C_10_H_14_N_5_O_7_P	347.221	Nucleotide and its derivates	0.59098	0.032129	Down
17	1.060	Borneol	C_10_H_18_O	154.249	Terpenoids	1.55483	0.044955	Up
18	6.040	Citroside A	C_19_H_30_O_8_	386.194	Terpenoids	2.00210	0.018877	Up
19	9.751	Longifolene	C_15_H_24_	204.350	Terpenoids	0.75707	0.048815	Down
20	9.970	Beta-Ionone	C_13_H_20_O	192.297	Terpenoids	0.82862	0.048116	Down
21	10.140	Elemol	C_15_H_26_O	222.198	Terpenoids	0.75625	0.023813	Down
22	10.795	Tanshinone II B	C_19_H_18_O_4_	310.347	Terpenoids	0.76223	0.021306	Down
23	0.880	N1-Acetylspermine	C_12_H_28_N_4_O	244.377	Amines	0.72696	0.008203	Down
24	4.430	N,N-Dimethyl-1,4-phenylenediamine	C_8_H_12_N_2_	136.194	Amines	2.43345	0.031564	Up
25	6.350	1-Decanoyl-2-hydroxy-sn-glycero-3-phosphocholine	C_18_H_38_NO_7_P	411.500	Amines	1.39888	0.045250	Up
26	9.540	4-D-Hydroxysphinganine	C_18_H_39_NO_3_	317.507	Amines	0.69954	0.003165	Down
27	0.800	Pyridoxamine	C_8_H_12_N_2_O_2_	168.090	Vitamins	1.59019	0.034441	Up
28	0.970	Pyridoxine di-O-hexoside	C_20_H_31_NO_13_	493.465	Vitamins	1.75554	0.034493	Up
29	1.050	Vitamin D2	C_28_H_44_O	396.648	Vitamins	1.35118	0.029204	Up
30	1.530	Pyridoxine	C_8_H_11_NO_3_	169.178	Vitamins	1.75554	0.018088	Down
31	5.100	N-Feruloyl putrescine	C_14_H_20_N_2_O_3_	264.100	Phenols and its derivatives	2.33433	0.017474	Up
32	6.490	Sinapaldehyde	C_11_H_12_O_4_	208.211	Phenols and its derivatives	0.53038	0.012306	Down
33	7.532	Ferulaldehyde	C_10_H_10_O_3_	178.185	Phenols and its derivatives	0.82819	0.020825	Down
34	9.209	Bornyl acetate	C_12_H_20_O_2_	196.290	Phenols and its derivatives	0.56864	0.005409	Down
35	4.910	N-p-Coumaroylputrescine	C_13_H_18_N_2_O_2_	234.290	Phenolic acids	2.26059	0.000463	Up
36	5.540	Hydroxycinnamate	C_9_H_8_O_3_	164.158	Phenolic acids	0.61848	0.045476	Down
37	11.630	5-O-Caffeoylshikimic acid	C_16_H_16_O_8_	336.293	Phenolic acids	1.72815	0.038816	Up
38	1.000	iP9G	C_16_H_23_N_5_O_5_	365.384	Phytohormones	2.28196	0.009327	Up
39	4.620	trans-zeatin N-glucoside	C_16_H_23_N_5_O_6_	381.200	Phytohormones	1.67891	0.024310	Up
40	1.540	Lupinine	C_10_H_19_NO	169.267	Alkaloids and derivatives	0.77060	0.043363	Down
41	2.834	Hordenine	C_10_H_15_NO	165.230	Alkaloids and derivatives	0.33202	0.028973	Down
42	0.680	Spermidine	C_7_H_19_N_3_	145.246	Polyamine	0.63059	0.035179	Down
43	4.285	5-Hydroxymethylfurfural	C_6_H_6_O_3_	126.110	Organooxygen compounds	0.60215	0.020847	Down
44	6.120	Sattabacin	C_13_H_18_O_2_	206.280	Benzene and substituted derivatives	1.85913	0.011933	Up
**ESI− mode**								
1	0.690	Thymidine 5′-diphosphate	C_10_H_16_N_2_O_11_P_2_	402.190	Nucleotide and its derivates	0.59820	0.009880	Down
2	0.730	dATP	C_10_H_16_N_5_O_12_P_3_	491.001	Nucleotide and its derivates	0.71596	0.019214	Down
3	0.980	2-deoxyglucose-6-phosphate	C_6_H_13_O_8_P	244.136	Nucleotide and its derivates	2.35486	0.000842	Up
4	4.289	Thymidine	C_10_H_14_N_2_O_5_	242.230	Nucleotide and its derivates	0.52871	0.041315	Down
5	1.038	Shikimic acid	C_7_H_10_O_5_	174.150	Phenolic acids	2.22761	0.008091	Up
6	5.801	Caffeic acid	C_9_H_8_O_4_	180.160	Phenolic acids	3.39126	0.004990	Up
7	6.040	2,6-Di-tert-butylphenol	C_14_H_22_O	206.320	Phenolic acids	1.98445	0.033550	Up
8	1.040	P-Coumaryl alcohol	C_9_H_10_O_2_	150.174	Phenylpropanoids and polyketides	1.36602	0.019139	Up
9	6.970	Oxyresveratrol	C_14_H_12_O_4_	244.240	Phenylpropanoids and polyketides	0.65530	0.029290	Down
10	0.950	Bisulfurous acid	C_11_H_8_O_2_	172.180	Vitamins	1.86849	0.017363	Up
11	5.920	3-O-p-coumaroyl shikimic acid O-hexoside	C_22_H_26_O_12_	482.100	Alcohols and polyols	1.74780	0.012347	Up

## Data Availability

The original contributions presented in the study are included in the article/Supplementary Materials. Further inquiries can be directed to the corresponding authors.

## Supplementary Materials

The following supporting information can be downloaded at https://www.mdpi.com/article/10.3390/foods14183283/s1, Figure S1. Representative total ion chromatograms of kale samples from control and selenate groups in positive ion mode. Figure S2. Representative total ion chromatograms of kale samples from control and selenate groups in negative ion mode.
