# Supplementary material for: Effect of Selenium Fortification on Growth Performance and Nutritional Compounds of Kale (Brassica oleracea L. Var. acephala DC.)
Source: Foods. 2025 Sep 22;14(18):3283. doi: 10.3390/foods14183283 (PMC12469545; doi:10.3390/foods14183283)
Supplement: Supplementary file 1 [file foods-14-03283-s001.zip › foods-3825057-supplementary.pdf]

(A)

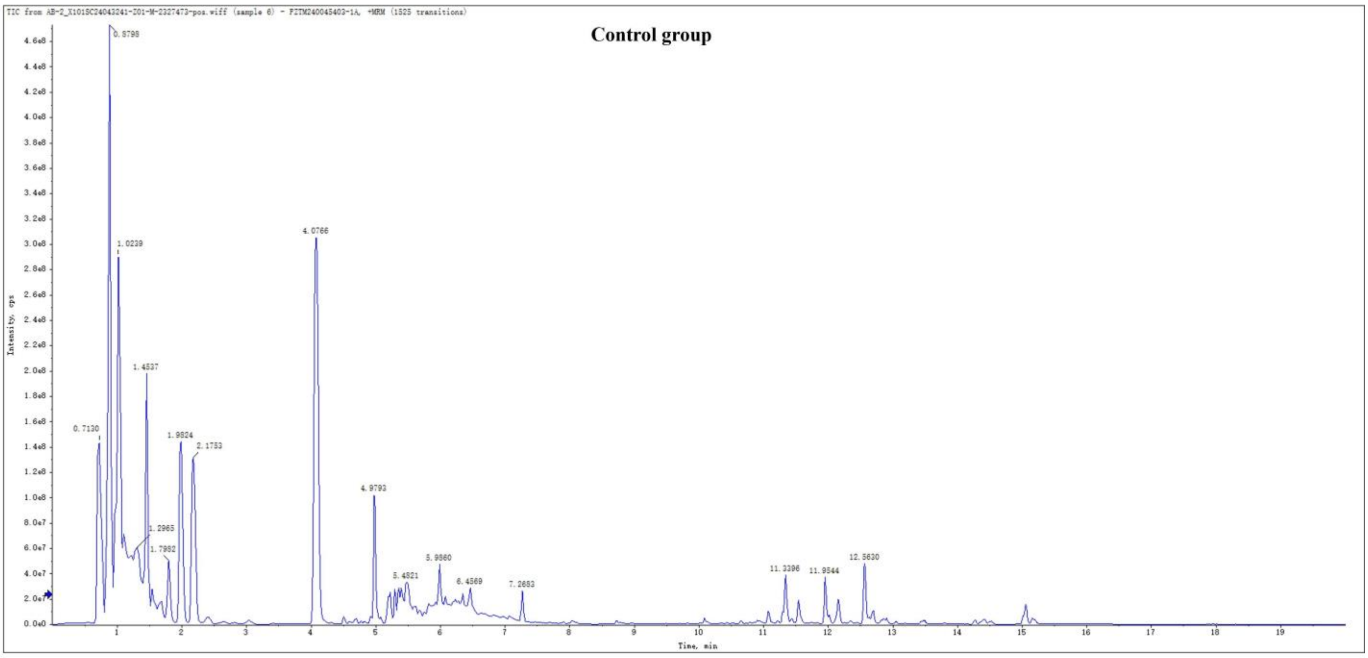

(B)

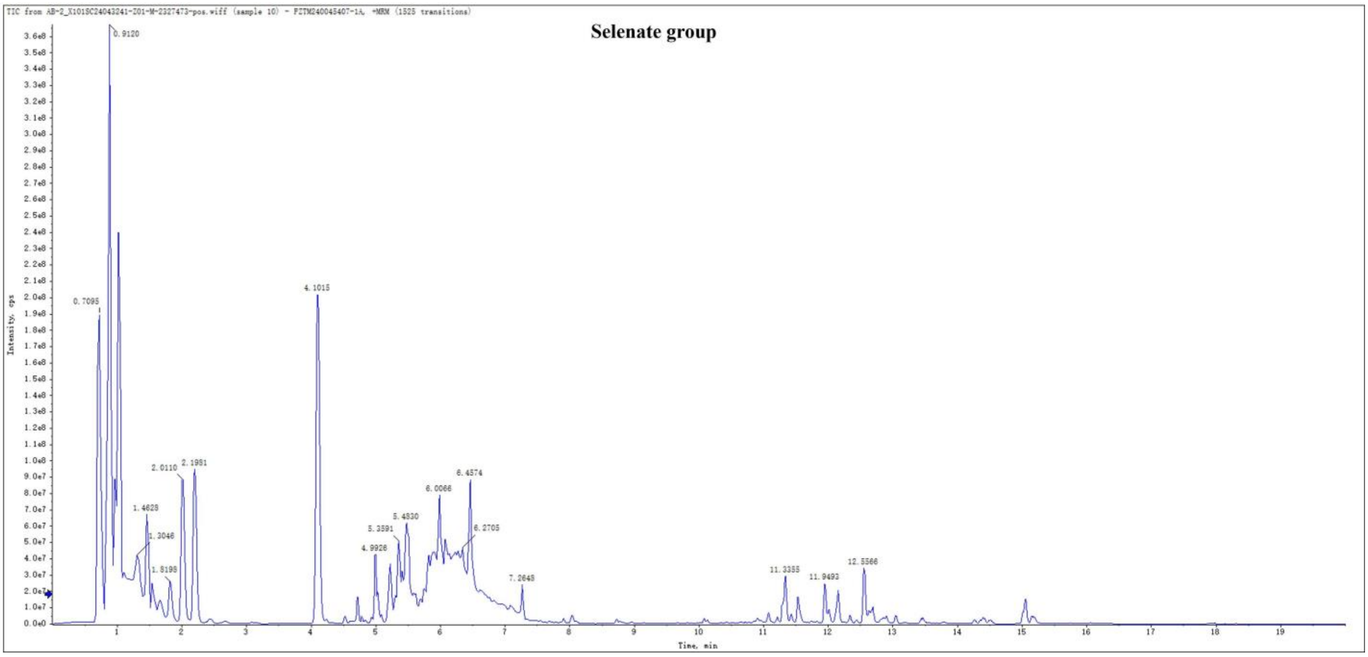

Figure S1 Representative total ion chromatograms of kale samples from Control and Selenate groups in positive ion mode.

(A)

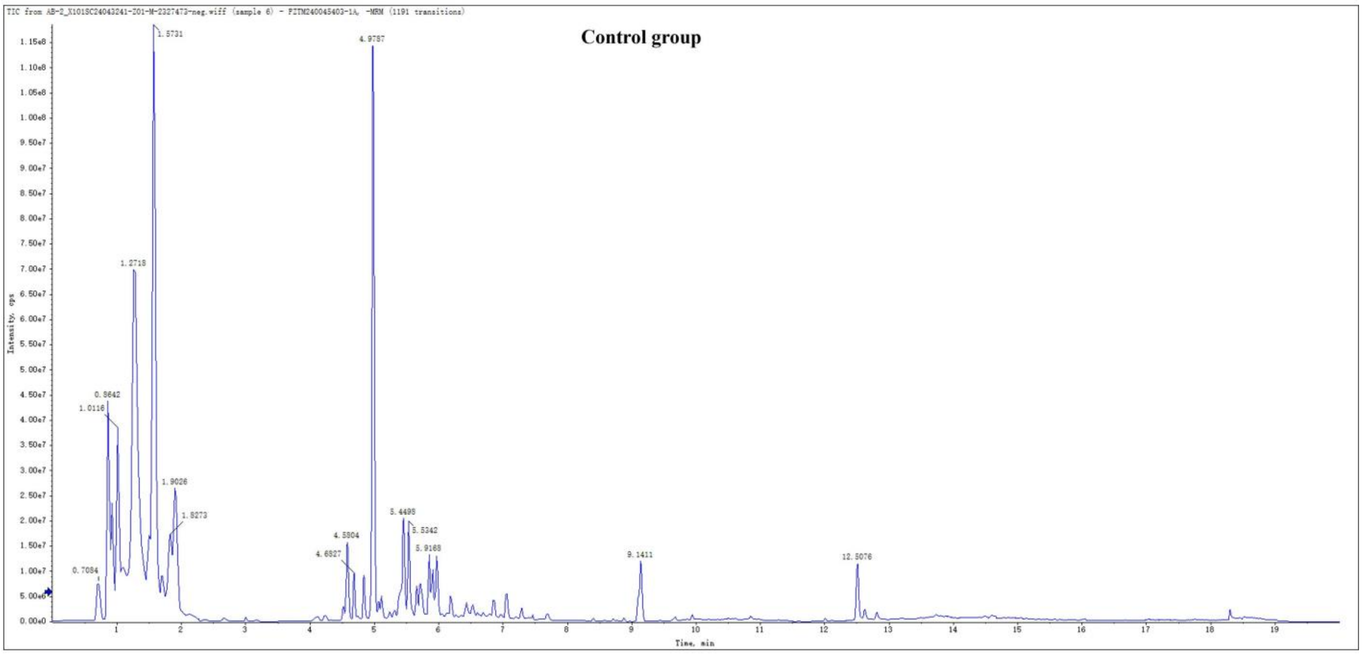

(B)

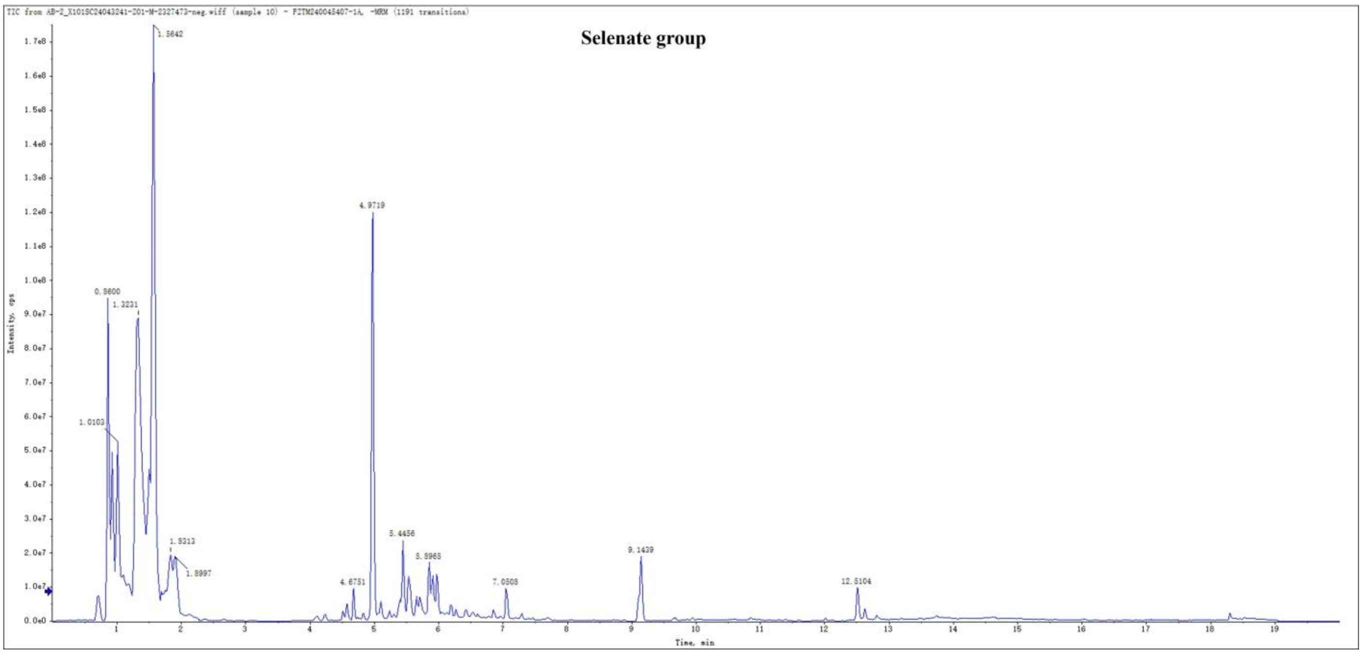

Figure S2 Representative total ion chromatograms of kale samples from Control and Selenate groups in negative ion mode.
